# Urban and rural habitats differ in number and type of bird feeders and in bird species consuming supplementary food

**DOI:** 10.1007/s11356-015-4723-0

**Published:** 2015-05-24

**Authors:** Piotr Tryjanowski, Piotr Skórka, Tim H. Sparks, Waldemar Biaduń, Tomasz Brauze, Tomasz Hetmański, Rafał Martyka, Piotr Indykiewicz, Łukasz Myczko, Przemysław Kunysz, Piotr Kawa, Stanisław Czyż, Paweł Czechowski, Michał Polakowski, Piotr Zduniak, Leszek Jerzak, Tomasz Janiszewski, Artur Goławski, Leszek Duduś, Jacek J. Nowakowski, Andrzej Wuczyński, Dariusz Wysocki

**Affiliations:** Institute of Zoology, Poznań University of Life Sciences, Wojska Polskiego 71C, 60-625 Poznań, Poland; Institute of Nature Conservation, Polish Academy of Sciences, Mickiewicza 33, 31-120 Krakow, Poland; High School of Civil Sciences, Zamojska 47, 20-1012 Lublin, Poland; Department of Vertebrate Zoology, Faculty of Biology and Environment Protection, Nicolaus Copernicus University, Lwowska 1, 87-100 Toruń, Poland; Department of Zoology, Pomeranian University, Arciszewskiego 22b, 76-200 Słupsk, Poland; Department of Zoology and Landscaping, University of Technology and Life Sciences, Ks. A. Kordeckiego 20, 85-225 Bydgoszcz, Poland; Przemysl Ornithological Society, Węgierska 6, 37-700 Przemyśl, Poland; Upper Silesian Ornithological Society, pl. Jana III Sobieskiego 2, 41-902 Bytom, Poland; Institute for Administration and Tourism, State Higher Vocational School in Sulechów, Armii Krajowej Str. 51, 66-100 Sulechów, Poland; Department of Environmental Protection and Management, Bialystok University of Technology, Wiejska 45a, 15-351 Białystok, Poland; Department of Avian Biology and Ecology, Faculty of Biology, Adam Mickiewicz University, Umultowska 89, 61-614 Poznań, Poland; Faculty of Biological Sciences, University of Zielona Góra, Prof. Z. Szafrana St. 1, PL 65-516 Zielona Góra, Poland; Department of Teacher Training and Biodiversity Studies, University of Łódz, Banacha 1/3, 90-237 Łódź, Poland; Department of Zoology, University of Natural Sciences and Humanities in Siedlce, Prusa 12, 08-110 Siedlce, Poland; Institute of Nature Conservation, Polish Academy of Sciences, Lower-Silesian Field Station, Podwale 75, 50-449 Wrocław, Poland; Department of Ecology and Environmental Protection, University of Warmia and Mazury in Olsztyn, PlacŁódzki 3, 10-727 Olsztyn, Poland; Department of Vertebrate Anatomy and Zoology, University of Szczecin, Wąska 13, 71-412 Szczecin, Poland

**Keywords:** Human-wildlife interaction, Human support, Supplemental food, Urbanization, Wintering, Urban ecosystems, Central Europe

## Abstract

Bird feeding is one of the most widespread direct interactions between man and nature, and this has important social and environmental consequences. However, this activity can differ between rural and urban habitats, due to inter alia habitat structure, human behaviour and the composition of wintering bird communities. We counted birds in 156 squares (0.25 km^2^ each) in December 2012 and again in January 2013 in locations in and around 26 towns and cities across Poland (in each urban area, we surveyed 3 squares and also 3 squares in nearby rural areas). At each count, we noted the number of bird feeders, the number of bird feeders with food, the type of feeders, additional food supplies potentially available for birds (bread offered by people, bins) and finally the birds themselves. In winter, urban and rural areas differ in the availability of food offered intentionally and unintentionally to birds by humans. Both types of food availability are higher in urban areas. Our findings suggest that different types of bird feeder support only those species specialized for that particular food type and this relationship is similar in urban and rural areas.

**Electronic supplementary material:**

The online version of this article (doi:10.1007/s11356-015-4723-0) contains supplementary material, which is available to authorized users.

## Introduction

Urbanization is increasing across the globe and it is recognized as a major factor affecting species, populations and assemblages (Turner et al. 2004; Grimm et al. 2008). Although urbanization is recognized as a major threat to biodiversity, there is increasing evidence that urban habitats may play a role in conservation (Jokimäki and Suhonen 1998; Chamberlain et al. 2004; Evans et al. 2009). However, the general belief is that more natural environments, such as rural areas, provide a more suitable habitat for most species and thus for their conservation (Turner et al. 2004; Evans et al. 2009). Indeed, human settlements in rural and urban areas differ in many structural and biotic components and even in human attitudes concerning wildlife (Clergeau et al. 1998; Lepczyk et al. 2004). The artificial structure of the urban ecosystem often results in inhabitants of towns and cities actively seeking contact with nature and wild animals (Savard et al. 2000), but in urban landscapes, the presence of wildlife is limited by the availability of habitats, human disturbance, collisions with vehicles and behavioural shyness (Fernandez-Juricic and Jokimäki 2001; Ditchkoff et al. 2006). However, various techniques are used to attract wildlife into urban areas. Among the many different methods to increase the number of wild animals in urban areas, supplementary food provision using bird feeders is probably the best known way to support birds in winter.

Human settlements may be especially favourable for birds during winter when climatic conditions are harsh and food is in poor supply. Urban areas are heat islands during winter with temperatures 1–2 °C higher than in the surrounding rural landscape (Rizwan et al. 2008; Jokimäki et al. 2009). Climatic conditions are one of the most important constraints in the distribution of bird species during winter (Newton 1998; Jokimäki et al. 2009). Therefore, an urban environment may favour bird species during this period. Urban areas usually have a low availability of natural food resources, implying that those species relying on these resources may be unwilling to use urban areas for wintering. However, supplementary feeding may reduce starvation and thus enhance winter survival in birds (Newton 1998), thus increasing the suitability of urban areas for birds. Since food is a major factor limiting bird populations (Newton 1998; Tratalos et al. 2007; Jokimäki et al. 2009), the effect of supplementary feeding should be most pronounced in a habitat where natural food resources are scarce (Chamberlain et al. 2009), such as in urban areas in comparison to more natural rural areas.

Bird feeding is one of the most widespread direct interactions between man and nature and has important social and environmental consequences (Tratalos et al. 2007; Robb et al. 2008a; Davies et al. 2012; Horn and Johansen 2013; Steyaert et al. 2014). Millions of people across the world participate in the feeding of wild birds, with almost half the households in many developed countries participating at a total cost of billions of US dollars annually (Orros and Fellowes 2012). Published studies indicate that winter feeding may positively affect bird populations by increased winter survival (Jansson et al. 1981; Brittingham and Temple 1988), enhanced breeding performance (Robb et al. 2008b; Wuczyński 2010) and predator avoidance (Dunn and Tessaglia 1994). However, negative effects on bird populations may include higher predation pressure reported in some studies (Dunn and Tessaglia 1994), transmission of parasites and disease (Brittingham and Temple 1988), lower egg quality (Plummer et al. 2013) and lower hatching success (Harrison et al. 2010). From a human perspective, bird feeding is very enjoyable (Lott 1988) but, at a more general scale, has cascading effects on ecosystems since studies have reported better control of plant parasites, such as aphids, in areas with bird feeders (Orros and Fellowes 2012).

Bird feeding interacts with processes of environmental change, thus creating a need for large-scale studies, with a particular focus on different habitats (Robb et al. 2008a). Feeders are, of course, directly associated with human settlements surrounded by habitats varying from rural to highly urbanized. Thus, the effects of bird feeding may be habitat-dependent and affect the structure of bird communities and, consequently, the urbanization processes in birds. One may predict that birds in an urban habitat with scarce natural food resources, such as plants or insect larvae, may be more dependent on feeders than in a rural habitat where natural food resources are more abundant, irrespective of the presence of bird feeders (Chamberlain et al. 2009). Thus, urban environments should be an environmental filter allowing only certain species to utilize these human-related food resources and achieve high densities.

However, supplementary feeding of birds takes various forms, a phenomenon not acknowledged in any previous studies. The location, size, form and other features of bird feeders may affect their exploitation by birds. Small feeders with a complicated structure and a small feeding area may only be used by small species, such as tits, and prevent larger species from foraging, eventually affecting species assembly structure and species interactions. Moreover, feeding may be supplemented with food not only in feeders but simply spread on the ground (a resource rarely quantified) or in litter bins allowing interspecific competition to play a major role in shaping bird communities at feeding areas. These different forms of supplementary feeding and their effect on wintering birds in different environments have not been previously investigated (Steyaert et al. 2014).

The aim of the current study was to examine differences in the value of human-provided food supplies for birds wintering in rural and urban habitats. We also investigate the importance of different types of bird feeder to particular bird species and discuss how this may potentially influence the number of species and individuals and even modify the urbanization process.

## Methods

### Study areas

We recorded the population density of wintering birds in 26 towns and cities (each paired with a nearby rural area) across Poland using the same methods (for more details, see Tryjanowski et al. 2015). The distance between urban and rural paired sites was 1–12 km.

### Field methods

Bird feeders were recorded by direct observation, located by following bird behaviour, as well as occasionally asking local people. They were counted within three 0.25-km^2^ squares (500 * 500 m) in each of the 26 urban areas in both December 2012 and January 2013. The same numbers of matched rural squares were recorded in the same period, to give a total of 156 squares in each month. Birds were observed during favourable weather conditions (no snow or rain, good visibility, wind below 4 m s^−1^) between 8:00 and 14:00 hours. Single observers walked through each square (60–90 min per square) to find all bird feeders, as well as all other potential food sources provided by humans. In each square, we noted the number of bird feeders both with and without food, the type of feeder (details below), additional food supplies potentially available to birds (e.g. bread offered by people, bins) and finally the birds themselves. To avoid disturbance, birds at feeders and those resting in the vicinity were counted from a distance. For each bird feeder, in both urban and rural squares, a control count in a similar habitat (but without available bird feeders) was also taken at a distance of 100–250 m from the feeder. At all points, birds were counted for a fixed time period of 5 min. This relatively short time was sufficient for the purposes of this study, since wintering birds exhibit a clumped distribution (i.e. with limited movements in order to save energy), bird detection was favoured by the transparency of winter habitats and by the fact that the birds wintering in settlements are accustomed to human presence. The short count time was balanced by the final large sample size (*n* = 1067) which was necessary to reliably compare bird occupancy at various types of feeders, control points and in two habitats (urban and rural).

**Fig. 1:**
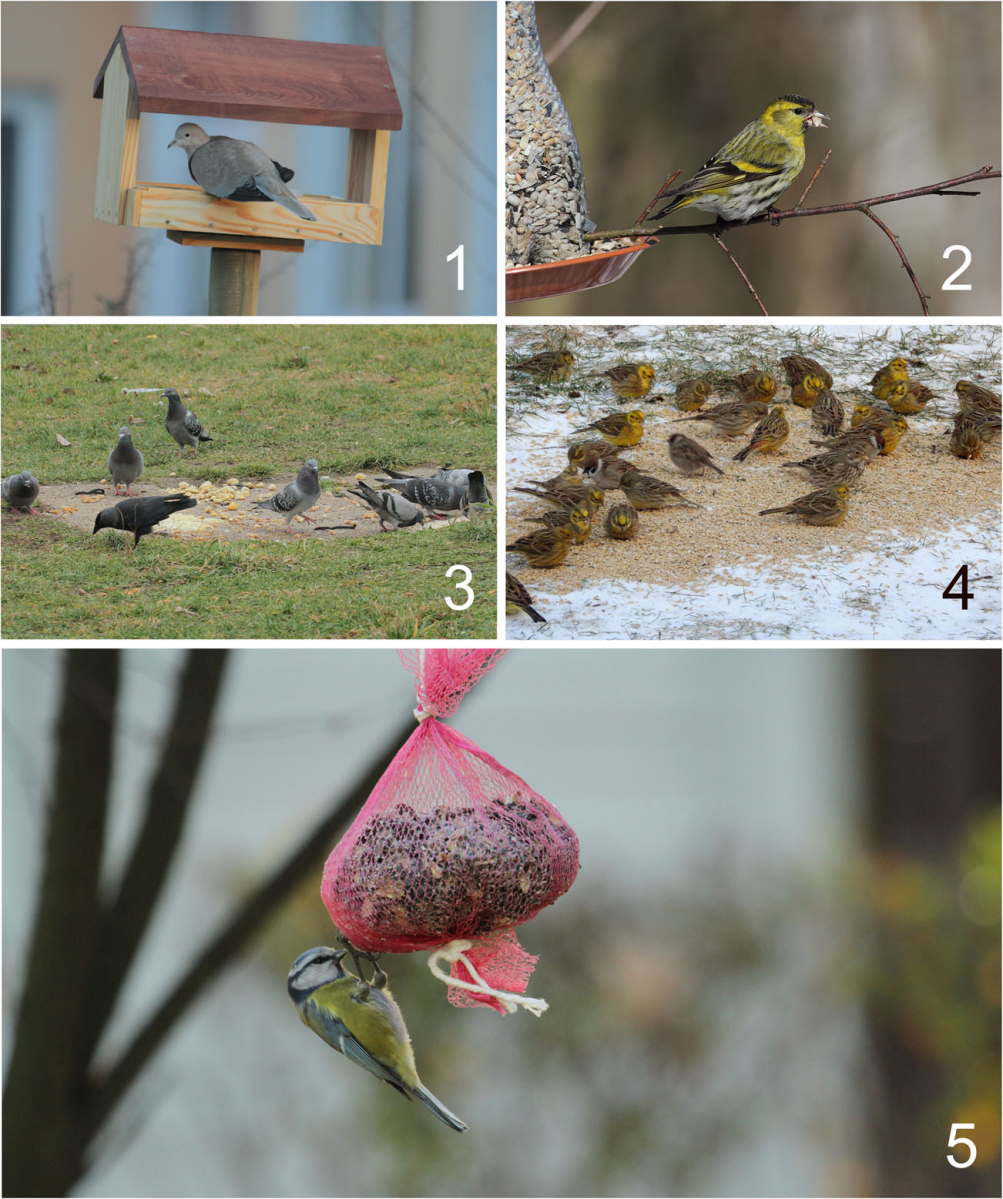
Examples of the following bird feeder categories: *1* typical bird table feeders with a roof; *2* automatic-type feeder providing mixed seeds; *3* waste human food, such as bread and boiled vegetables on the ground; *4* seeds, mainly wheat and sunflower, placed on the ground; *5* pig fat mixed with some seeds and prepared as a ball (authors of the pictures: S. Czyż, A. Graclik, M. Dobrzyńska and M. Stawowy)

Bird feeders were divided into five categories: (1) typical bird table feeders with a roof providing different types of food, mainly seeds; (2) automatic, mainly bottle-type feeder providing mixed seeds; (3) waste human food, such as bread and boiled vegetables provided usually on the ground; (4) seeds, i.e. mainly wheat and sunflower, placed on the ground or balcony; and (5) pig fat and/or skin sometimes mixed with some seeds and prepared as a block or ball (Fig. 1).

### Data analysis

Descriptive statistics were carried out using the MINITAB v.16 package. A chi-squared contingency table was used to test differences in the proportions of types of bird feeder in urban and rural areas. An ordination of mean numbers for the five types of feeder and for the controls in both urban and in rural areas (6 (types + control) × 2 habitat type combinations × 17 species) was undertaken in the CANOCO package. A principal components analysis (PCA) was carried out due to a short gradient length of 1.9. This analysis was prepared for the 17 species with more than 100 individuals observed during the study.

## Results

### Number of bird feeders and feeding sites with supplementary food

**Fig. 2:**
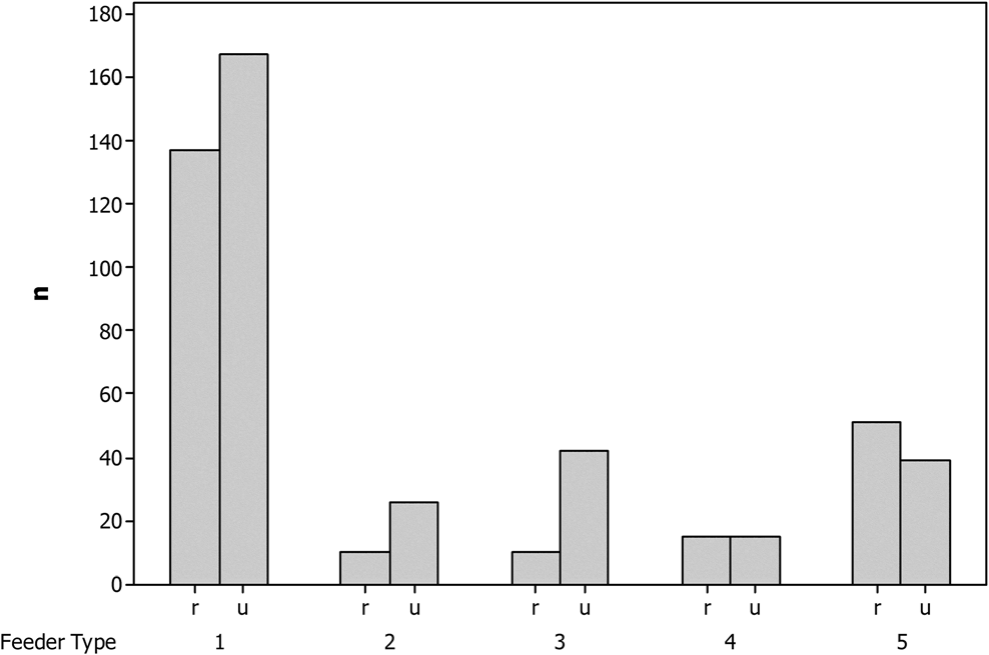
Distribution of types of bird feeder expressed as numbers (*n*) in rural (*r*) and urban (*u*) areas. *1* Typical roofed bird tables, *2* automatic seed feeder, *3* waste food, *4* seeds on the ground, *5* animal fat. For more details, see the “Methods” section

Urban and rural areas differed significantly in the numbers of feeders available to birds (Table 1); however, the proportion of bird feeders with food available was similar in both areas. Moreover, the numbers of particular types of bird feeder differed significantly between urban and rural areas (Fig. 2; *χ*^2^ = 23.24, df = 4, *P* < 0.001). In particular, urban-rural differences in feeder types 2 (automatic seed feeder), 3 (waste food) and 5 (animal fat) contributed to the large chi-squared value, while there was little difference in other feeder types (Fig. 2).

**Table 1:** A comparison of bird feeder information between urban and rural areas

Variable	Urban	Rural	*t* value	*P*
No. of bird feeders	15.91 ± 0.61	8.97 ± 0.61	−0.760	<0.001
No. of bird feeders with food	7.89 ± 0.38	4.41 ± 0.38	−3.430	<0.001
% of bird feeders with food	54.86 ± 1.79	51.12 ± 1.80	−0.483	0.143
No. of bins	5.92 ± 0.28	1.10 ± 0.28	−6.266	<0.001
No. of other supplementary food places	1.19 ± 0.09	0.51 ± 0.09	−3.146	<0.001

Data are presented as mean ± SE. Sample size is 78 squares (3 plots in each among 26 cities and towns, and accompanied rural habitats)—study plots for both urban and rural areas

### Birds

A total of 27,217 individual birds (18,900 at bird feeders, 8317 at control points) were recorded from 51 species (44 and 45 at bird feeders and control points respectively) (Table A.1). The ten most common species in decreasing order of abundance were the following: house sparrow *Passer domesticus*, feral pigeon *Columba livia* var. *urbana*, great tit *Parus major*, rook *Corvus frugilegus*, jackdaw *Corvus monedula*, tree sparrow *Passer montanus*, greenfinch *Chloris chloris*, collared dove *Streptopelia decaocto*, blue tit *Cyanistes caeruleus* and magpie *Pica pica*.

**Fig. 3:**
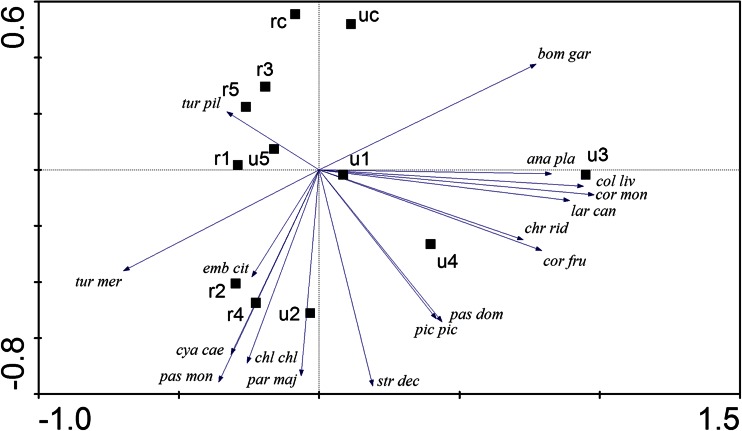
Principal component biplot showing the relationships between the 17 most common bird species (see Online Resource 1 for the species abbreviations) with combinations of rural (*r*) and urban (*u*) areas and bird feeders: *1* typical roofed bird tables, *2* automatic seed feeder, *3* waste food, *4* seeds on the ground, *5* animal fat, *c* control points provided in both habitat types

Birds were associated with particular types of bird feeder and control points, and the first two axes of the PCA explained 60.4 % of their variability (PC1 = 37.9 %, PC2 = 22.5 %), and both ordination axes were statistically significant at *P* < 0.05. A biplot of the PCA is shown in Fig. 3. In broad terms, species to the right of the ordination had higher densities in urban habitats. Species towards the bottom of the ordination are those more associated with seed feeders (u2, u4, r2, r4). There is some suggestion that the influence of urban feeders may be greater than their rural equivalents, for example the dominant position of u3 on the first axis.

Waxwing *Bombycilla garrulus* was the most numerous species associated with the urban habitat, while the characteristic species for rural areas was fieldfare *Turdus pilaris*.

## Discussion

Even as the world becomes increasingly urbanized and interconnected, a distinction between urban and rural areas still exists (Lepczyk et al. 2004; Tratalos et al. 2007; Møller et al. 2012). Around the world, different groups of people have varying levels of exposure to natural hazards and gradual climatic change, as well as access to different coping and resiliency strategies that create unique sets of assets and vulnerabilities (Lott 1988; Lepczyk et al. 2004).

Bird feeding by humans differed between urban and rural areas for several reasons. Human population size and the structure of dwellings, for example big blocks of flats only occurred in cities, affect the potential number of interactions between humans and birds. The economic status of people can also be important. Rural areas are rather poorer and thus they provide cheaper bird food, such as animal fat. In contrast, more expensive bird food is provided in the cities where humans tend to be more affluent (Steyaert et al. 2014). Moreover, in cities, the waste food of schools, restaurants, supermarkets and similar places is being utilized by birds (Robb et al. 2008a; Skórka et al. 2009; Oro et al. 2013). We also showed clear and significant differences in the number of bird feeders and number of bird feeders with food, both of which were higher in cities than in rural areas. This is potentially related to the number of inhabitants, but Davies et al. (2012) also suggested other associations, such as the age of the head of the household and annual income.

We showed that bird feeders are attractive places for birds and probably support as many as 65 % of individual wintering birds in urban and rural areas (cf. data for all species in Supplementary Material). This is in agreement with many previous studies, which suggested that bird feeders can change and modify winter avifauna (Cowie and Hinsley 1988; Robb et al. 2008a, b; Jones 2011). As far as we are aware, our results are the first evidence that these modifications can vary between rural and urban areas and are related to differences in the type of bird feeder and also in other sources of food, such as bins and waste food provided unintentionally by humans. The results obtained from an ordination analysis suggest that rural and urban areas are similar in the availability of food from bird feeders, suggesting that bird feeders are an important factor affecting winter bird communities, perhaps even more than other environmental variables (Cowie and Hinsley 1988; Strohbach et al. 2009; Oro et al. 2013). Potentially, therefore, feeders and other supplementary food may play a role in the further urbanization processes of birds (Anderies et al. 2007; Chamberlain et al. 2009; Oro et al. 2013). Some species more commonly winter in rural areas while others favour urban areas. This is well known for large species, such as gulls, ducks and corvids, for which cities provide a lot of options in winter (Sorace 2002; Maciusik et al. 2010; Polakowski et al. 2010; Tryjanowski et al. 2015), but for more farmland birds, such as yellowhammer, finches, or tree sparrow, rural areas with farmland patches are the more important habitat (e.g. Ciach 2012; Villén-Pérez et al. 2013; Tryjanowski et al. 2015).

Paradoxically, animal fat appeared to be associated with fieldfare, but this species occurs more often in rural habitats where people hang fat and skin on fruit trees which also happen to be an important place for wintering thrushes (Skórka et al. 2006). In this context, why the waxwing was the only common species seemingly not affected by bird feeders is clear, since in winter this species is associated mainly with the fruits of ornamental trees which are more numerous in cities (Strohbach et al. 2009; Laband et al. 2013). They only rarely use supplementary food provided directly by humans.

Obviously, the use of different bird feeders is probably caused by the different food provided in particular feeder types. Other factors such as vulnerability to predators (e.g. related to feeder location and height above the ground—Villén-Pérez et al. 2013), and even small differences in thermopreferences (Villén-Pérez et al. 2013) may play an important role. However, it is not possible to distinguish between these factors without undertaking a manipulative experimental study.

Because the bird feeding market is still increasing (Robb et al. 2008a; Oro et al. 2013), we believe these findings have important implications for future studies of urbanization and for invasion biology in general. Different food and bird feeder types in both urban and rural environments may mediate species winter survival and species interactions and thus affect bird communities.

## Conclusions

In this large-scale study conducted in 26 locations across Poland (156 squares of 0.25 km^2^), we showed marked differences between urban and rural areas regarding supplementary feeding of wintering birds. Despite the high scale of bird feeding in both areas, the intensity of feeding and the frequency of five types of feeders were different. Much higher number of feeders and other supplementary food sources were recorded in urban areas confirming greater supplementary food available to birds in the cities. Consequently, twice as many wintering birds were noted in urban compared to rural areas. Moreover, more than twice as many individuals were associated with supplementary feeding locations compared to control locations, both in urban and rural areas. Finally, the composition of bird communities was affected by supplementary feeding. Although species richness was similar in both environments, community composition varied according to the type of feeders; for example, larger species (gulls, corvids) were particularly associated with waste food in the cities and not in the rural areas.

These data strengthen the general conclusion that artificial food provisioning has enormous ecological impacts, affecting the number, distribution and behaviour of birds during winter. However, we documented clear differences in bird feeding between urban and rural habitats and interesting patterns in bird responses to these activities. These differences are likely directly linked to various lifestyles and other drivers of human society, such as economic status, attitude towards wildlife and education. Populations of wild birds are therefore under various pressures in response to changing urbanization gradients; however, the long-term effects of these interactions need further understanding.

## Electronic supplementary material

Online Resource 1 Species recorded during the study, species codes (used in ordination) and total numbers of birds recorded at rural and urban feeders and controls. Species are arranged in order of descending overall abundance. (RTF 165 kb)
