# Supplementary material for: Urban and rural habitats differ in number and type of bird feeders and in bird species consuming supplementary food
Source: Environ Sci Pollut Res Int. 2015 May 24;22(19):15097–103. doi: 10.1007/s11356-015-4723-0 (PMC4592493; doi:10.1007/s11356-015-4723-0)
Supplement: Supplementary file 1 — Species recorded during the study, species codes (used in ordination) and total numbers of birds recorded at rural and urban feeders and controls. Species are arranged in order of descending overall abundance. (RTF 165 kb) [file 11356_2015_4723_MOESM1_ESM.rtf]

Article title:
Urban and rural habitats differ in number and type of bird feeders and birds using supplementary food

Journal name:
ENVIRONMENTAL SCIENCE AND POLLUTION RESEARCH

Autor names:
Piotr Tryjanowski et al.

Affiliation and e-mail address of the corresponding author:
Piotr Tryjanowski, Institute of Zoology, Poznañ University of Life Sciences, Wojska Polskiego 71C, PL-60-625 Poznañ, Poland; piotr.tryjanowski@gmail.com

Online Resource 1 
Species recorded during the study, species codes (used in ordination), and total numbers of birds recorded at rural and urban feeders and controls. Species are arranged in order of descending overall abundance.
species	code	rural	rural	urban	urban	Total	
		feeders	control	feeders	control		
Passer domesticus	pas dom	2200	543	3000	970	6713	
Columba livia f. urbana	col liv	7	89	2449	1590	4135	
Parus major	par maj	1130	303	1766	382	3581	
Corvus frugilegus	cor fru	110	156	1239	999	2504	
Corvus monedula	cor mon	42	51	1074	1003	2170	
Passer montanus	pas mon	873	138	468	99	1578	
Chloris chloris	chl chl	584	106	610	61	1361	
Streptopelia decaocto	str dec	183	114	353	115	765	
Cyanistes caeruelus	cya cae	245	63	262	83	653	
Pica pica	pic pic	120	118	232	166	636	
Turdus merula	tur mer	181	103	183	104	571	
Chroicocephalus ridibundus	chr rid	0	1	293	162	456	
Bombycilla garrulus	bomgar	0	102	83	112	297	
Anas platyrhynchos	ana pla	0	3	255	0	258	
Turdus pilaris	tur pil	92	38	37	35	202	
Larus canus	lar can	0	0	96	96	192	
Emberiza citrinella	emb cit	133	41	13	0	187	
Coccothraustes coccothraustes	coc coc	44	8	31	13	96	
Fringilla coelebs	fri coe	43	12	35	4	94	
Garrulus glandarius	gar gla	43	30	14	5	92	
Carduelis cannabina	car can	3	82	3	0	88	
Columba palumbus	col pal	0	0	57	19	76	
Carduelis spinus	car spi	34	21	3	17	75	
Periparus ater	per ate	40	11	3	0	54	
Larus argentatus	lar arg	0	0	22	28	50	
Erithacus rubecula	eri rub	28	3	7	4	42	
Corvus cornix	cor cornix	5	5	17	12	39	
Poecile palustris	pae pal	30	3	0	0	33	
Carduelis carduelis	car car	7	4	10	11	32	
Pyrrhula pyrrhula	pyr pyr	7	15	8	2	32	
Dendrocopos major	den maj	10	11	1	2	24	
Sitta europaea	sit eur	10	2	9	1	22	
Regulus regulus	reg reg	9	3	7	1	20	
Sturnus vulgaris	stu vul	2	1	13	1	17	
Lophophanes cristatus	lop cri	4	3	4	1	12	
Aegithalos caudatus	aeg cau	0	1	0	9	10	
Accipiter nisus	acc nis	1	3	1	2	7	
Corvus corax	cor cor	0	6	0	0	6	
Certhia brachydactyla	cer bra	1	1	1	2	5	
Dendrocopos syriacus	den syr	0	0	3	2	5	
Troglodytes troglodytes	tro tro	3	1	1	0	5	
Dendrocopos medius	den med.	1	2	0	0	3	
Fringilla montifringilla	fri mon	3	0	0	0	3	
Galerida cristata	gal cri	0	1	0	2	3	
Phasianus colchicus	pha col	3	0	0	0	3	
Accipiter gentilis	acc gen	1	0	1	0	2	
Certhia familaris	cer fam	1	0	0	1	2	
Poecile montanus	pae mon	2	0	0	0	2	
Picus viridis	pic vir	0	0	1	1	2	
Falco tinnunculus	fal tin	0	0	0	1	1	
Turdus viscivorus	tur vis	0	0	0	1	1	
		6235	2198	12665	6119	27217	
